# Nail in the appendix: Asymptomatic foreign body lodging detected by imaging

**DOI:** 10.1016/j.radcr.2025.10.020

**Published:** 2025-11-11

**Authors:** Husam Abdulsattar Alhasan, Kyle Sheets, Rajendra Kedar

**Affiliations:** a University Medical Center Goettingen (UMG), Robert-Koch-Str. 40, Goettingen 37075, Germany; b Department of Radiology, University of South Florida Morsani College of Medicine, 2 Tampa General Circle, Tampa, FL 33606, USA

**Keywords:** Foreign body, Nail ingestion, Appendix, Computed tomography, Serial radiographs, Appendectomy

## Abstract

Foreign body ingestions are relatively common and often pass through the gastrointestinal tract without complications. Asymptomatic lodging of a foreign body within the appendix, particularly in adults, is exceedingly rare and has only been described in a handful of cases. Ingestion of sharp or elongated objects, however, can result in impaction, perforation, or other complications. We present the case of a 55-year-old female who accidentally ingested a metallic nail, which ultimately lodged in her appendix. Despite being completely asymptomatic, serial radiographs and computed tomography confirmed the position of the nail in the right lower quadrant. The patient underwent elective laparoscopic appendectomy, and histopathological examination revealed active appendicitis. This case illustrates the importance of serial imaging, particularly CT, in tracking sharp foreign bodies and detecting silent complications.

## Introduction

Foreign body ingestion is a common clinical scenario, particularly in children, psychiatric patients, and individuals in institutional settings. Most ingested objects pass through the gastrointestinal tract without incident, with less than 1% requiring surgical intervention. Common sites of impaction include anatomical narrowings such as the upper esophagus at the cricopharyngeus muscle, the pylorus, and the ileocecal valve [1].

Appendiceal lodging of foreign bodies is exceedingly rare, occurring in fewer than 0.0005% of all ingestion cases [2,3]. In the literature, such cases are frequently discovered incidentally postoperatively or on imaging [4]. Radiographic visibility depends on the material. Radiopaque objects like metal are typically visible on plain films, while radiolucent materials may require computed tomography (CT) or ultrasound [5,6]. CT is particularly valuable in identifying complications such as perforation, especially in cases involving sharp or elongated foreign bodies [6,7].

## Case report

A 55-year-old female with no significant past medical or surgical history presented to the emergency department after accidentally ingesting a nail while hanging pictures. She had been holding the nail between her teeth, looked upward, and unintentionally swallowed it. She reported no abdominal pain, nausea, vomiting, or other symptoms. Physical examination was unremarkable, and laboratory values were reported as normal.

**Fig. 1:**
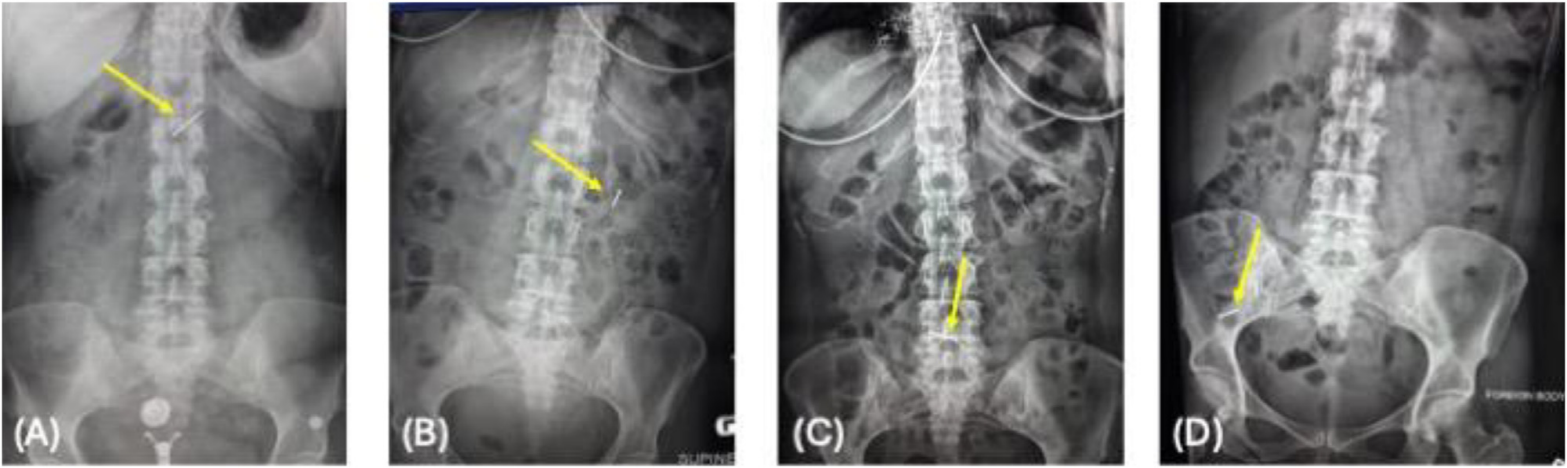
(A) Abdominal X-ray showing a linear radiopaque object in the epigastric region. (B) Follow-up X-ray showing the object in the left upper quadrant. (C) Further progression of the object into the lower abdomen. (D) Radiograph showing the object in the right lower quadrant. Yellow arrows indicate the object in all images.

**Fig. 2:**
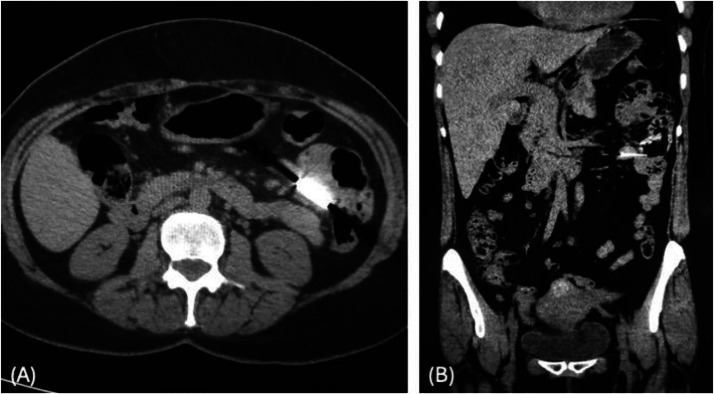
(A) Axial non-contrast CT of the abdomen showing a dense linear object within the small bowel in the left upper quadrant. (B) Coronal CT image confirming intraluminal location in the small intestine.

At initial presentation in the emergency department, a plain abdominal radiograph showed a 3.0 × 0.3 cm metallic foreign body in the epigastric region (Fig. 1A). Esophagogastroduodenoscopy was performed soon after, but the object could not be visualized; the esophagus, stomach, and duodenum appeared normal. Subsequently, a non-contrast CT scan of the abdomen and pelvis revealed the nail within the small bowel in the left upper quadrant, without evidence of perforation (Figs. 2A and B). A repeat abdominal radiograph obtained shortly after confirmed the same position (Fig. 1B).

Imaging demonstrated progressive movement of the nail through the gastrointestinal tract, and by the next day it had advanced toward the right lower quadrant (RLQ) (Figs. 1C and D).

**Fig. 3:**
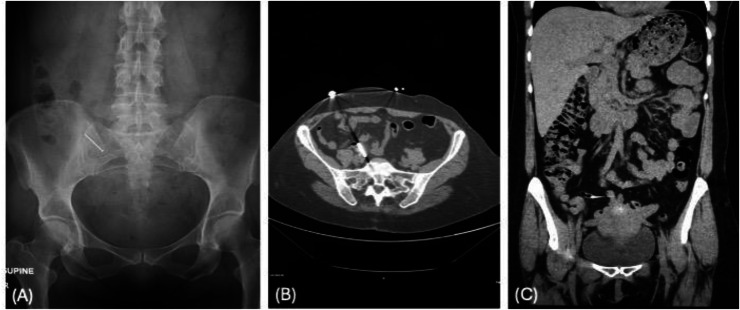
(A) Abdominal X-ray obtained three weeks after ingestion, showing the object persisting in the right lower quadrant. (B) Axial CT image showing the object within the appendix. (C) Coronal CT image confirming intra-appendiceal location without radiologic signs of inflammation or perforation.

Three weeks after the initial ingestion, the patient returned for reevaluation. She remained asymptomatic and reported no changes in bowel habits. A follow-up X-ray demonstrated that the nail was still located in the RLQ (Fig. 3A). A non-contrast CT scan localized the nail to the appendix (Figs. 3B and C), without mural thickening, fat stranding, or appendiceal dilatation.

**Fig. 4:**
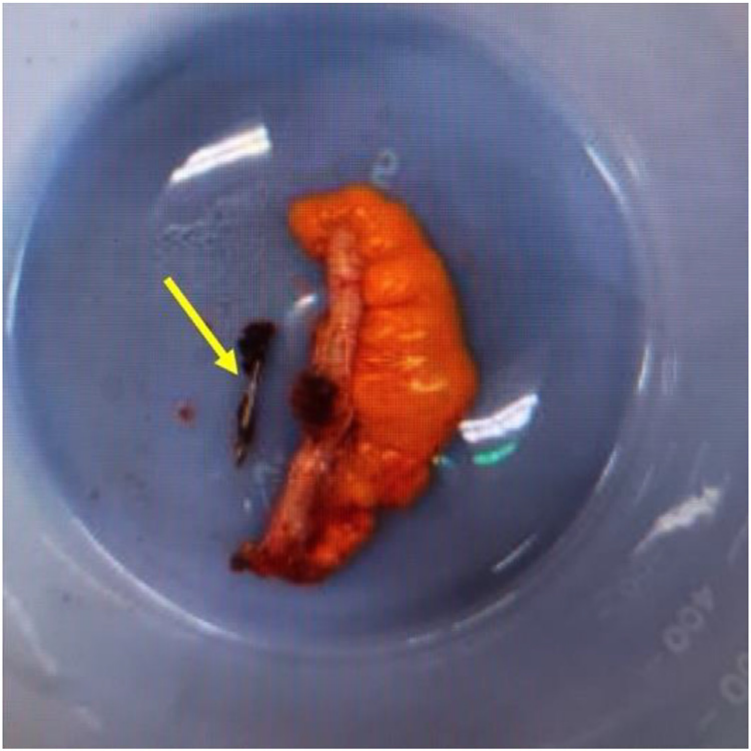
Intraoperative photograph of the excised appendix with the metallic nail placed adjacent to the specimen. A yellow arrow indicates the nail, which measured 2.6 × 0.1 cm.

Despite the lack of symptoms, the decision was made to proceed with an elective laparoscopic appendectomy to prevent possible complications. Intraoperatively, the appendix appeared normal, and the nail was palpated inside its lumen. The nail was confirmed within the excised specimen (Fig. 4). Histologic examination revealed a 2.6 × 0.1 cm pointed, metallic foreign body and active appendicitis with eosinophilic infiltration, consistent with a localized inflammatory response to the foreign body rather than parasitic disease. No signs of perforation, neoplasia, or atypia were present.

## Discussion

Foreign body ingestion is most frequently encountered in children, with the majority of objects passing through the gastrointestinal tract without the need for intervention. Surgical removal is usually reserved for high-risk cases involving sharp, long, or impacted objects, particularly if they fail to progress or pose a risk for perforation [5]. While the appendix is an unusual location for a foreign body to lodge, particularly in adults, its occurrence has been estimated at fewer than 0.0005% of all ingestion cases. Most reported patients develop symptoms or are diagnosed incidentally after surgery, making truly asymptomatic appendiceal foreign bodies in adults exceptionally rare [2,3].

A recently published case involving a 3-year-old child described an incidental finding of a metallic object lodged in the appendix that remained in place over three months before being surgically removed [2]. Pediatric cases like that one highlight how ingestion is far more common in younger populations, likely due to behavioral tendencies. In contrast, our case demonstrates that the same complication can occur in completely asymptomatic adults and may go unrecognized without consistent radiologic follow-up. The management strategies may also differ between pediatric and adult patients. In children, longer periods of observation are often considered unless the object is high-risk, whereas in adults the persistence of a foreign body, particularly in the appendix, more strongly favors surgical removal to prevent perforation or delayed appendicitis [2,3,5].

The choice of imaging modality depends largely on the material composition of the ingested object. Metallic items like nails are radiopaque and readily visible on plain radiographs, as was the case here. However, CT was still necessary in our patient to precisely localize the object within the appendix and to exclude subtle complications. In contrast, other materials such as plastic or wood may not be consistently detected without advanced imaging techniques like CT or ultrasound. Table 1 summarizes the radiographic visibility of commonly ingested materials and illustrates the importance of tailoring imaging modality choice to the suspected object type [[5], [6], [7]]. This quick reference is particularly useful in emergency settings, where knowledge of material-dependent visibility can guide efficient and accurate imaging selection.

**Table 1:** Radiographic visibility of commonly ingested materials.

Material type	Radiographic visibility	Preferred imaging/notes
Metal	Radiopaque	Easily detected on plain X-ray
Glass	Radiopaque	Visible on X-ray
Stone	Radiopaque	Visible on X-ray
Wood	Radiolucent	Often missed; CT or US needed
Plastic	Variable	CT or US recommended
Fish bone	Often radiolucent	CT preferred over X-ray

Our patient remained symptom-free despite the nail remaining lodged in the appendix for over three weeks. The foreign body was visualized throughout via serial abdominal X-rays and later confirmed by CT. Although conservative management can be considered in asymptomatic individuals, the risk of delayed appendicitis or perforation, especially with sharp, metallic objects, supports elective surgical removal when radiologic evidence shows persistent localization in the RLQ [2,3,8]. Current guidelines recommend removal of sharp or elongated foreign bodies that fail to progress, given their potential for impaction or perforation [1]. In the appendix, the narrow lumen predisposes to stasis, and reported cases consistently show high rates of appendicitis or perforation when sharp objects are left in place [3,8]. For this reason, surgical intervention is generally favored even in asymptomatic patients once localization to the appendix is confirmed. If surgical removal had been deferred, potential complications described in the literature include acute appendicitis, perforation with peritonitis, intraabdominal abscess, fistula formation, and sepsis [4,8]. Histopathologic analysis in our case confirmed active appendicitis, reinforcing the clinical significance of proactive imaging and intervention.

## Conclusion

This case adds to the limited literature on foreign bodies lodged in the appendix and underscores the importance of considering this diagnosis not only in pediatric but also in adult patients. Even in the absence of symptoms, persistent localization of a sharp foreign body on imaging should prompt surgical evaluation, as serious pathology may otherwise go unnoticed. A key radiology teaching point is that radiologists should consider appendiceal lodging of sharp foreign bodies in cases where RLQ localization persists on serial imaging, even in asymptomatic adults.

## Patient consent

The author(s) should confirm that written informed consent has been obtained from the involved patient(s) or if appropriate from the parent, guardian, power of attorney of the involved patient(s); and, they have given approval for this information to be published in this case report (series).

## Author contributions

Husam Abdulsattar Alhasan: Conceptualization, Investigation, Data curation, Writing - Original draft, Visualization. Kyle Sheets: Conceptualization, Investigation, Writing - Review & Editing. Rajendra Kedar: Supervision, Writing - Review & Editing.
